# Clinical significance of serum IGFBP‐3 in colorectal cancer

**DOI:** 10.1002/jcla.22912

**Published:** 2019-06-19

**Authors:** Yu‐Lei Hou, Peng Luo, Guang‐yan Ji, Hui Chen

**Affiliations:** ^1^ Clinical Laboratories The First Affiliated Hospital of Chongqing Medical University Chongqing China; ^2^ Department of Gastrointestinal Surgery The First Affiliated Hospital of Chongqing Medical University Chongqing China

**Keywords:** colorectal cancer, diagnostic efficacy, serum IGFBP‐3

## Abstract

**Background:**

IGF‐binding protein 3（IGFBP‐3）has previously been identified as tumor marker. The present study aimed to investigate the clinical significance of serum IGFBP‐3 in colorectal cancer (CRC).

**Methods:**

Serum was collected from 70 CRC patients and 50 healthy volunteer controls. Serum IGFBP‐3 and carcinoembryonic antigen (CEA) levels were measured using electrogenerated chemiluminescence immunoassay and compared between groups. Relationships between serum IGFBP‐3 level and the clinical characteristics of CRC were also analyzed. A receiver operator characteristic (ROC) curve was plotted to investigate diagnostic efficacy of serum IGFBP‐3 and CEA, respectively, for CRC. Data were analyzed using SPSS 13.0.

**Results:**

Serum IGFBP‐3 levels in CRC were lower than those of controls (4.68 [3.56, 5.77] vs 5.44 [4.77, 6.10] µg/mL, *P* < 0.05). Furthermore, serum IGFBP‐3 levels were higher in early cancer stages (I and II) than those of advanced stages (III and IV) (4.78 [3.92, 5.49] vs 3.77 [2.65, 4.59] µg/mL, *P* < 0.05). In addition, patients with lymph node metastasis absent had elevated serum IGFBP‐3 levels than those of patients with lymph node metastasis present (4.73 [3.92, 5.72] vs 4.11 [2.45, 4.83] µg/mL, *P* = 0.02). Finally, ROC curve indicated that serum IGFBP‐3 had a better diagnostic power for CRC than CEA. When serum IGFBP‐3 and carcinoembryonic antigen were used together to detect CRC, the AUC was 0.949, with a sensitivity of 75% and a specificity of 90%.

**Conclusions:**

Serum IGFBP‐3 might be a potential biomarker for CRC.

## 1 INTRODUCTION

Colorectal cancer (CRC) is the third most common cancer, with a global estimated mortality of more than 600 000 deaths each year.^1^ If CRC is detected at a localized stage, 5‐year patient survival is 90%.^2^ Thus, a more efficient diagnostic marker for the early detection of CRC would greatly improve survival. Colonoscopy, the standard screening method for CRC, is invasive and inconvenient, while an alternative detection method, the fecal occult blood test, lacks sufficient sensitivity and specificity.^3^ Existing serum biomarkers for CRC also lack sufficient sensitivity and specificity. Consequently, sensitive, specific, and noninvasive serum markers for CRC are urgently required.

The insulin‐like growth factor (IGF) pathway plays important roles in human cancer development through apoptosis suppression and promotion of cell cycle progression.^4^ IGFBP‐3 as the main binding molecule of IGF plays a critical role in regulating IGF function.^5^ The relationships between IGFBP‐3 and some cancers have been reported. Serum IGFBP‐3 levels in lung cancer patients were found to be lower than those of controls;^6^ however, in breast cancer, IGFBP‐3 expression induced mammary cell growth.^7^ Tas also reported that serum IGFBP‐3 might be a complement marker for CA125 in epithelial ovarian cancer diagnosis.^8^ In addition, IGFBP‐3 expression was found to be lower in pancreatic cancer patients than that of controls.^9^ However, the relationship between serum IGFBP‐3 and CRC has not been reported yet.

Previous reports have indicated that the IGF system played an important role in CRC cell proliferation.^10^, ^11^ However, the clinical significance of serum IGFBP‐3 level in CRC patients has not yet been reported. The present study aimed to measure serum IGFBP‐3 levels in CRC patients and investigate the clinical significance of serum IGFBP‐3 in CRC.

## 2 MATERIALS AND METHODS

### 2.1 Subjects

A total of 70 patients with untreated CRC (34 male, 36 female, age 62.3 ± 5.8 years) were identified from the Department of Gastrointestinal Surgery at the First Affiliated Hospital of Chongqing Medical University, China, between January and April 2017. Serum was collected from patients and 50 controls (25 male, 25 female; age 62.1 ± 5.3 years). Each patient underwent colon resection, and the CRC diagnosis was confirmed by histological examination. The identified patients and controls provided written consent to participate in the study, and the protocol was approved by the Ethics Committee of the First Affiliated Hospital of Chongqing Medical University. Tumor staging was evaluated according to the 6th edition of the International Union Against Cancer: Tumor‐Node‐Metastasis Classification for malignant tumors.^12^

### 2.2 Sample collection

Venous blood (2.0 mL) was collected from each subject into tubes without anticoagulant. Serum was separated by centrifugation at 2000 *g* for 10 minutes, and samples were stored at −80°C until use.

### 2.3 Serum IGFBP‐3 and CEA measurement

Serum IGFBP‐3 levels were measured using chemiluminescence immunoassay (Immulite 1000, Siemens Healthcare Diagnostics Inc,), and serum CEA levels were measured using electrogenerated chemiluminescence immunoassay (Cobas E602, Roche Diagnostics).

### 2.4 Statistical analysis

The normality of data was checked by the Kolmogorov‐Smirnov test, and the data were not normally distributed. Serum IGFBP‐3 levels were summarized as median (interquartile range). Results were compared between groups using the Mann‐Whitney *U* test. The relationships between serum IGFBP‐3 and CEA were investigated by using binary logistic regression analysis. A receiver operating characteristic (ROC) curve was plotted to determine the area under the curve (AUC), sensitivity, and specificity of serum IGFBP‐3 and CEA levels for CRC, respectively. Statistical analysis was performed using SPSS 13.0 software (Chicago, IL, USA), and *P* < 0.05 represented a statistically significant result.

## 3 RESULTS

### 3.1 Patient clinical characteristics

A total of 70 CRC patients were enrolled in the study. The clinical characteristics are summarized in Table 1. Those characteristics included gender, the information of CRC patients with diabetes mellitus (DM), TNM stage, and the status of lymph node metastasis and tumor differentiation and are listed in Table 1. As DM, there are six patients (8.6%) with DM and 64 patients (91.4%) without DM. As TNM stage, 28 patients (65.1%) were in early stages (I and II) and 15 patients (34.9%) were in advanced stages (III and IV). As the status of lymph node metastasis, there were 40 patients (57.1%) with lymph node metastasis absent and 30 patients (42.9%) with lymph node metastasis present.

**Table 1:** Clinical characteristics of colorectal cancer patients

Characteristic	n (%)
Gender,(female/male)	34/36 (48.6%/51.4%)
Age, ($\overset{¯}{x}\pm s$)	62.3 ± 5.8
Patients with diabetes mellitus	
Yes	6(8.6%)
No	64(91.4%)
TNM stage^a^	
Early stages (I and II)	28 (65.1%)
Advanced stages (III and IV)	15(34.9%)
Lymph node metastasis	
Absent	40 (57.1%)
Present	30(42.9%)
Tumor differentiation^a^	
Well	10(19.6%)
Moderate	36(70.6%)
Poor	5(9.8%)

a Some data missing.

As tumor differentiation, there were 10 patients (19.6%) with well differentiation, 36 patients (70.6%) with moderate differentiation, and five patients (9.8%) with poor differentiation. Furthermore, the difference in serum IGFBP‐3 levels was not found between CRC patients with and patients without DM (3.83 [3.33，5.31] vs 4.72 [3.60，5.77] µg/mL, *P* = 0.456).

### 3.2 Serum IGFBP‐3 and CEA levels in CRC patients

**Figure 1:**
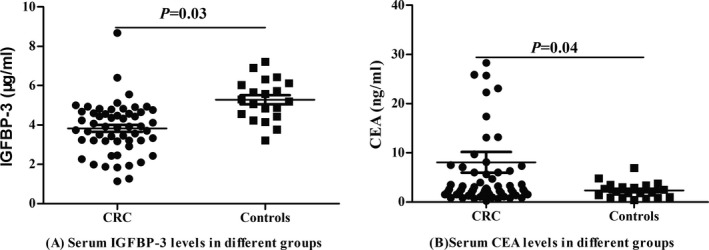
Comparisons of serum IGFBP‐3 and CEA levels in CRC and controls

Serum IGFBP‐3 levels in CRC patients were significantly lower than those of controls (4.68 [3.56, 5.77] vs 5.44 [4.77, 6.10] µg/mL, *P* = 0.03; Figure 1A); serum CEA levels in CRC patients were higher than those of controls (2.6 [1.6, 6.3] vs (1.8 [1.4, 2.5] ng/mL, *P* = 0.04; Figure 1B).

### 3.3 Relationships between serum IGFBP‐3 and CRC clinical characteristics

Patients with early stages (I and II) and lymph node metastasis absent showed higher serum IGFBP‐3 levels than those of patients with advanced stages (III and IV) and lymph node metastasis present (4.78 [3.92, 5.49] vs 3.77 [2.65, 4.59] µg/mL, *P* = 0.04; (4.73 [3.92, 5.72] vs 4.11 [2.45, 4.83] µg/mL, *P* = 0.02), while no significant associations were found between CEA levels and clinical characteristics(2.2 [1.5, 6.2] vs 2.5 [1.8, 4.1] ng/mL, *P* = 0.931; 2.2 [1.5, 6.1] vs 3.0 [2.1, 6.3] ng/mL, *P* = 0.35).

**Figure 2:**
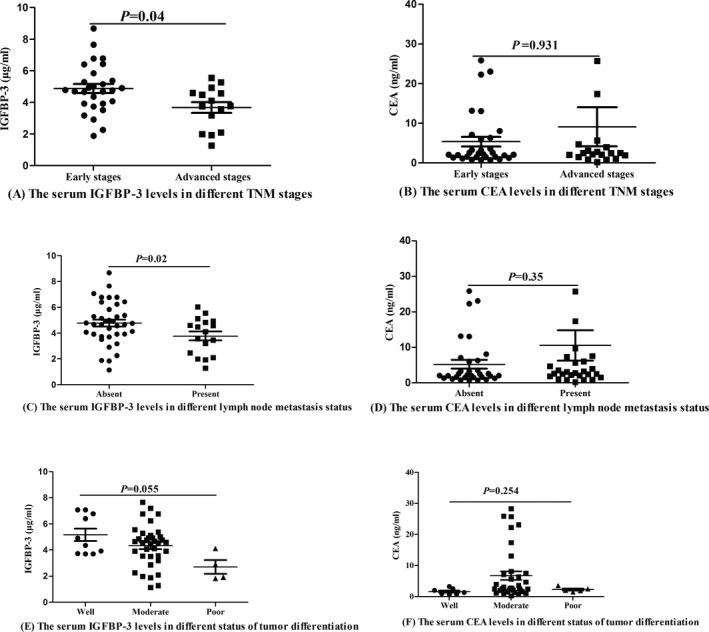
Relationships between serum IGFBP‐3 and colorectal cancer clinical characteristics

The serum IGFBP‐3 levels were 4.63 (3.79, 6.63) µg/mL, 4.59 (3.5, 5.03) µg/mL, and 2.42 (1.90, 3.20) µg/mL in the different groups of CRC patients with well differentiation, moderate differentiation, and poor differentiation, respectively. Although the statistical difference in serum IGFBP‐3 levels was not found between different tumor differentiation (*P* = 0.055), an interesting trend was appeared that the lower the serum IGFBP‐3 levels, the poorer the differentiation. But the statistical difference and trend of serum CEA levels between different tumor differentiation were not found (1.30 [0.87, 2.25] ng/mL vs 2.60 [2.02, 7.20] ng/mL vs 2.0 [1.90, 2.50] ng/mL, *P* = 0.254). Figure 2.

### 3.4 Relationships between serum CEA and IGFBP‐3

**Figure 3:**
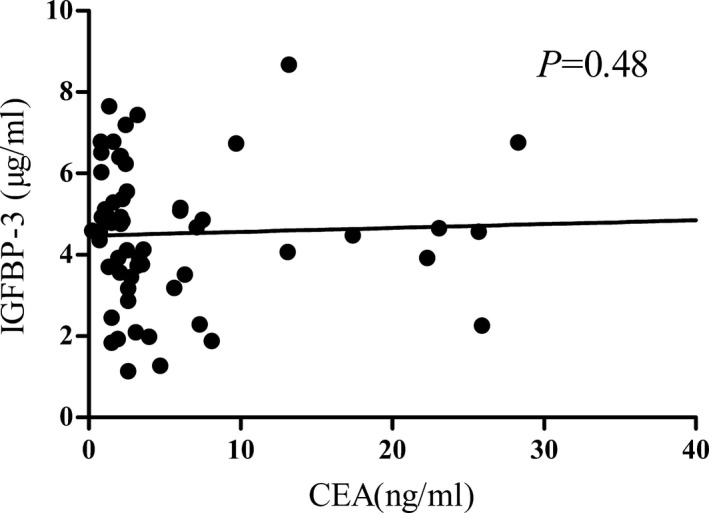
Relationships between serum carcinoembryonic antigen and IGFBP‐3

No significant associations were found between serum IGFBP‐3 and CEA levels (*r* = 0.092, *P* = 0.48; Figure 3). The result indicated that serum IGFBP‐3 might be a complementary marker for CEA, especially for patients with normal serum CEA levels.

### 3.5 Diagnostic efficacy of serum IGFBP‐3 for CRC

**Figure 4:**
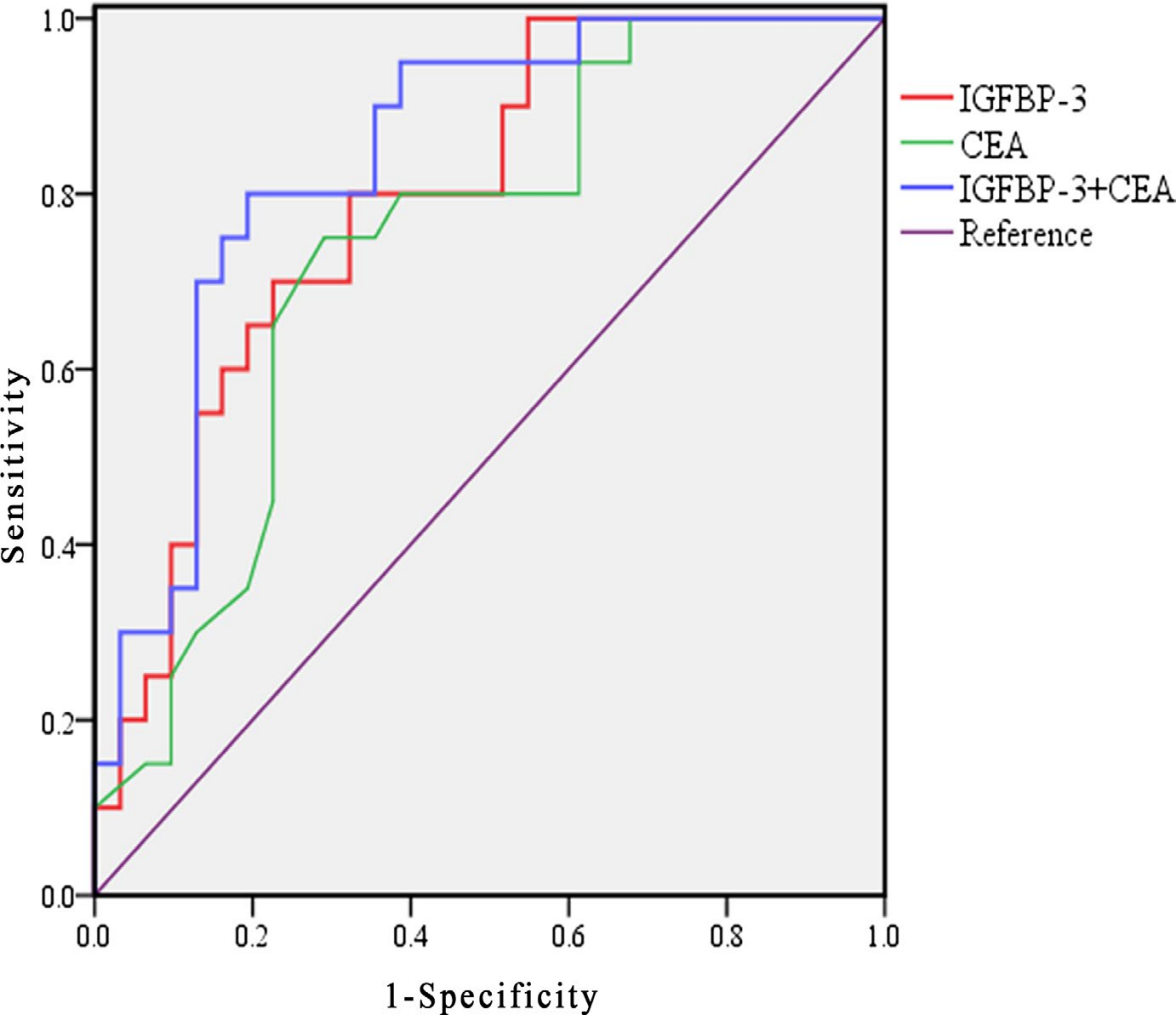
Comparisons of diagnostic efficacy of serum IGFBP‐3 and CEA levels for colorectal cancer

The ROC curve for serum IGFBP‐3 level in CRC (Figure 4) had an AUC of 0.826 (95% confidence interval [CI]: 0.721‐0.931). Youden’s index was calculated to set an optimum cutoff value of 4.84 µg/mL with a sensitivity of 70% and a specificity of 85.5%. The ROC curve of serum CEA level for CRC had an AUC of 0.757 (95% CI: 0.633‐0.881) with a sensitivity of 60% and a specificity of 80%. When serum IGFBP‐3 and CEA were combined for CRC detection, the AUC was 0.842, with a sensitivity of 75% and a specificity of 90%.

## 4 DISCUSSION

CRC is one of the most frequently diagnosed types of cancer. Previous studies have indicated that an increased risk of CRC might be associated with insulin‐like growth factors.^13^, ^14^, ^15^ Previous reports indicated that IGF system played an important role in the pathological process of cancer.^16^ IGFBP‐3 as a member of IGF system has been demonstrated to play an important role in various cancers, including non–small‐cell lung cancer, hepatocellular carcinoma, ovarian cancer, breast cancer, and gastric cancer.^17^ Yan et al^18^ found that IGFBP‐3 expression levels were lower in hepatocellular carcinoma tissues than those of adjacent nonmalignant liver tissue and low IGFBP‐3 levels were associated with high cancer risk, poor prognosis, and tumor metastasis in esophageal cancer.^19^ Keku et al^20^ reported that IGFBP‐3 gene expression was significantly lower in CRC tissue than that of normal colorectal tissue. Similarly, the present study indicated that serum IGFBP‐3 was lower in CRC patients than that of controls. All these studies indicated that serum IGFBP‐3 might be a potential biomarker for CRC.

Most CRCs are first diagnosed at an advanced stage in most cases. A better biomarker was expected to be closed with clinical characteristics. Previous reports indicated that higher IGFBP‐3 levels were closely associated with earlier clinical stages in esophageal squamous cell carcinoma.^21^ Yan et al found that in HCC tissue, IGFBP‐3 expression levels were inversely correlated with tumor size, node metastasis, and clinical stage.^18^ Zhao et al reported that the clinical pathological stage of esophageal cancer was inversely associated with serum IGFBP‐3 levels.^22^ Keku found that IGFBP‐3 mRNA levels were positively associated with apoptosis in CRC tissue, but in normal colonic tissue, no significant association was found between IGFBP‐3 mRNA levels and apoptosis.^20^ Our study indicated that serum IGFBP‐3 level was negatively correlated with CRC tumor stage and lymph node metastasis. Although the statistical difference in serum IGFBP‐3 levels was not found between different tumor differentiation (*P* = 0.055), an interesting trend was appeared that the lower the serum IGFBP‐3 levels, the poorer the differentiation. While no statistical difference and trend of serum CEA levels between different tumor differentiation were found, results of the present study are in accordance with the literature that IGFBP‐3 is associated with tumor clinical characteristics.

CEA, a tumor marker, has been widely used for CRC diagnosis in clinical practice.^23^ However, CEA expression has been found to be increased in certain noncancerous conditions, such as ulcerative colitis.^24^ The serum CEA level frequently increases several months before CRC recurrence and has been widely used as a marker for predicting CRC recurrence.^25^ But some patients showed high serum CEA levels despite the absence of CRC recurrence, while in others the serum CEA level is positive at initial surgery but becomes negative at the time of recurrence.^26^ Thus, CEA alone may not be a reliable marker for CRC detection and prognosis. Interestingly, the present study indicated that serum IGFBP‐3 level had better diagnostic power than serum CEA level in detecting CRC. Furthermore, when investigating the relationships between serum CEA and IGFBP‐3 levels, no relationships were found and the results indicated that serum IGFBP‐3 might be a complementary marker for CEA in CRC diagnosis. We found a combined ROC analysis of the use of both markers, and both sensitivity and specificity were relatively high when discriminating CRC from controls, indicating that the combination of serum CEA and IGFBP‐3 may be a useful tool in CRC detection.

In conclusion, serum IGFBP‐3 levels in CRC were significantly lower than those in controls and were closely correlated with CRC stage and lymph node metastasis. ROC analysis indicated that a combination of serum IGFBP‐3 and CEA level is a potential tool for CRC.

## CONFLICT OF INTEREST

The authors declare that they have no competing interests.
